# Improving feeding skills and transition to breastfeeding in early preterm infants: a randomized controlled trial of oromotor intervention

**DOI:** 10.3389/fped.2023.1252254

**Published:** 2023-09-18

**Authors:** Nilay Comuk Balci, Sahin Takci, H. Canan Seren

**Affiliations:** ^1^ Department of Physiotherapy and Rehabilitation, Faculty of Health Sciences, Ondokuz Mayıs University, Samsun, Türkiye; ^2^ Department of Neonatology, Faculty of Medicine, Ondokuz Mayıs University, Samsun, Türkiye

**Keywords:** preterm, oral-motor therapy programme, oral feeding, breast feeding, oral motor assessment scale

## Abstract

**Introduction:**

Oromotor therapy exercises used for preterm infants in the NICU might promote oral-motor skills and shorten discharge day. This study investigates the impact of an oral-motor therapy program on the successful transition to breastfeeding (BF) and the enhancement of feeding skills in preterm infants below 30 weeks of gestational age who experience feeding intolerance.

**Methods:**

The intervention group received oral-motor therapy programme for one month, while the control group did not. The feeding skills were evaluated by Early Feeding Skills Assessment Tool (EFS) and Preterm Oral Feeding Readiness Scales (POFRAS).

**Results:**

There was a significant difference in EFS and POFRAS scores, transition to bottle feeding at discharge and transition to BF after discharge between babies given oral-motor therapy programme and controls (*p* < 0.05). While the transition time to full enteral feeds did not vary significantly between the groups, noteworthy outcomes were observed in the intervention group, including differences in feeding type at discharge, the nature of feeds at discharge, and the success of transitioning to breastfeeding after discharge.

**Discussion:**

We conclude that the oromotor therapy exercises in NICU improves the quality of sucking, contributes to better oromotor skills and promotes transition to enteral feeding and BF in preterm babies.

**Clinical Trial Registration:**

ClinicalTrials.gov, identifier (NCT05845684).

## Introduction

Early preterm infants encounter many challenges across various physiological systems in the neonatal intensive care unit (NICU). Among these challenges, a notable difficulty involves attaining effective oral feeding skills and transitioning to breastfeeding. Initially, parenteral nutrition is initiated due to the infants' limited ability to tolerate enteral feeds and to promote adequate nutrition (1–3). Early implementation of enteral nutrition, accompanied by the administration of parenteral nutrition, has been shown to effectively mitigate growth retardation and enhance mental developmental scores in infants. Once enteral feeding is well-tolerated, a prompt transition to full enteral feeding is implemented (4). Immaturity of the gastrointestinal tract, reduced gastrointestinal motility, increased susceptibility to necrotizing enterocolitis (NEC), and other prematurity-related co-morbidities are the barriers to the transition of enteral feeding. Also, early preterm infants may face delays in oral feeding due to insufficient sucking and swallowing coordination, weak oropharyngeal musculature, and a higher likelihood of experiencing feeding aversions (5–7).

Until now, physical therapy in the NICU mainly included respiratory system exercises for infants and removing secretions as well as postural care. Nowadays, one of the physiotherapist's role is developing oral motor skills of the infants. Oromotor skills may enhance by oromotor therapy that focuses on strengthening the muscles involved in oral functions, such as sucking, swallowing, and breathing. By targeting these critical skills, oromotor therapy aims to improve the coordination and efficiency of feeding, ultimately facilitating successful breastfeeding and decreasing the day of NICU stay. There are no known side effects or complications of oro-motor therapy (8–11).

Previous studies have demonstrated that oromotor stimulation had benefits leads to varying conclusions in the management of premature infants (12). Our study aims to investigate the impact of oromotor exercise therapy administered in the NICU on the proficiency of transitioning to breastfeeding and improving feeding skills in early preterm infants experiencing feeding intolerance.

## Materials and methods

### Procedure

This randomized-controlled study encompassed preterm infants born at 30 weeks of gestation or earlier, with a birth weight under 1,500 g, and have feeding intolerance who were admitted to the Neonatal Intensive Care Unit at Ondokuz Mayıs University Health Practice and Research Center from January 2022 to April 2023. Feeding intolerance, a gastrointestinal disorder, primarily affects premature infants and is characterized by gastric residuals, abdominal distension, and/or vomiting. This condition often leads to extended hospitalization in NICUs (13).

**Figure 1:**
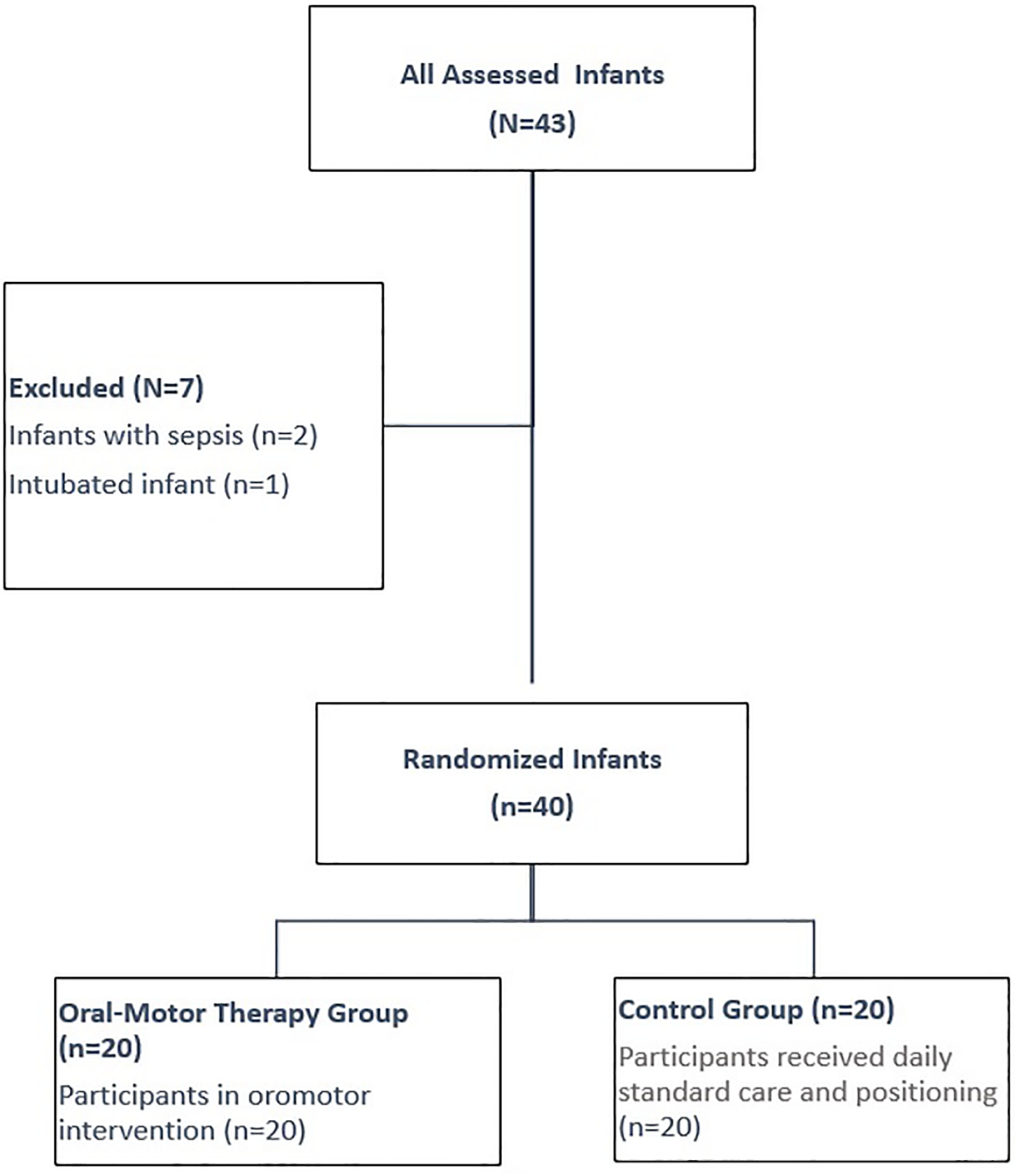
Flow diagram of the current study.

Preterm infants followed in the NICU and whose vital signs were stable were assigned to two groups, as oromotor intervention group and the control group. The intervention commenced once the infant's vital signs stabilized, around the 31th week of post-conceptional age. A centralized web-based randomization system was used for randomization. The infants were assigned to either oromotor therapy group or control group (Figure 1). The randomization sequence was generated utilizing the computerized R program (version 3.5.1. software).

Infants with major congenital abnormalities, invasive mechanical ventilation, sepsis and NEC were excluded from the study. Oral-motor therapy group received 15–20-min sessions 3 days a week for 1 month, massage of the mouth and swallowing muscles, tactile stimuli around the mouth and in the mouth to stimulate sucking, and non-nutritive sucking exercises. Daily standard care and supine, prone and side lying positions were applied to the control group.

All procedures were conducted in accordance with the principles outlined in the Declaration of Helsinki. Additionally, all protocols were approved by the Ethics Committee of Ondokuz Mayis University (2021/608). The registration number for this RCT in the Clinical Trial Registration was NCT05845684.

Infants included in the oral-motor therapy program were evaluated twice, just before the therapy started and after the therapy program. After obtaining the demographic information of the baby and parents in the first evaluation, the vital signs of the baby were recorded; In the second evaluation phase, after one month of oral-motor therapy, the infant's feeding skills were evaluated with the Early Feeding Skills Assessment Tool (EFS) scale and the Preterm Oral Feeding Readiness Scale (POFRAS). We recorded time to transition to full enteral feeding, duration of hospital stay, breastfeeding and nutritional outcomes, feeding method, diet type, and milk intake. We made the same evaluation procedure for the control group. After the vital signs were stable of the infants in the control group we waited for one month and made EFS and POFRAS evaluations for each baby. In the control group, the infants did not receive oro-motor therapy, and only got daily standard care and positioning. There were 20 subjects in each goup and no dropouts occurred in any of the groups.

#### NICU feeding and discharge protocol

In the NICU, mothers were informed about the importance of breastfeeding and encouraged to express their breast milk during their infant's NICU stay via breast pumps located in the NICU and at home. Minimal enteral nutrition was started for the infants as soon as they were hemodynamically stable, and enteral feeding was advanced according to the unit protocol. Kangaroo care was performed as soon as the infant was stable. The infants were first fed by orogastric (OG) tubes. Non-nutritive sucking (NNS) was endorsed in all infants, especially when the infant is on nasal Continuous Positive Airway Pressure (CPAP). Oral feeding was offered to the infant after the postconceptional 33rd weeks of gestation. Earlier, brief trials of breastfeeding were possible when the infant was on kangaroo care. Infants were discharged when they weighed more than 2,000 g, without apnea and desaturation during the last week, caffeine therapy had stopped, the infant was fully enterally fed, and the mother-infant dyad had adapted to each other. Infants and mothers were kept together for at least two days in special adaptation rooms before discharge from the NICU. If the infant was ready for discharge but cannot get all his feeding via the oral route, mothers are taught how to apply an OG tube to the infant during their stay in the adaptation room.

#### Evaluation of sociodemographic status and nutritional performance

Sociodemographic information of the infants (sex, gestational age, postnatal age, APGAR score, birth weight, mode of delivery, maternal birth information and parents' age, occupation, and educational status) was recorded. Vital signs (oxygen saturation, blood pressure, heart rate), and changes in height-weight information of the baby were recorded, and growth and development were followed. Information about the infant's feeding performance, feeding type, feeding time, amount of milk intake, time of full enteral feeding, and daily weight gain were recorded.

#### Early feeding skills assessment tool (EFS)

In 2005, Thoyre, Shaker, and Pridham developed the EFS (Feeding Skills Evaluation) to assess the feeding skills of infants during the transition to oral feeding (13). Girgin et al. (14). created Turkish version of the EFS. The EFS comprises 19 items divided into 5 subscales: respiratory regulation, oral-motor function, swallowing coordination, engagement, and physiologic stability. The tool facilitates the assessment of preterm infants' readiness for oral feeding and their oral feeding skills. It also enables the observation of symptoms linked to problematic feeding, helping in the planning of targeted feeding interventions to address areas where the infant encounters challenges or needs support during the transition to oral feeding. The evaluation of items pertaining to feeding skills utilizes a 3-option structure: “skill not yet observed” (1 point), “skill emerging” (2 points), and “skill consistently observed” (3 points). Indicators of problems within a skill are scored using a frequency-based 3-option structure: frequent indication of a problem (1 point), the occasional indication of a problem (2 points), and never or rare indication of a problem (3 points). The overall EFS score is calculated as the sum of the 5 subscale scores, ranging from 19 to 57. Higher scores on the scale reflect more mature feeding skills (15).

#### Preterm oral feeding readiness assessment scale (POFRAS)

POFRAS, developed by Fujinaga et al. (16), is a tool designed to assess readiness for oral feeding in preterm infants. It comprises 18 items organized into 5 categories: corrected gestational age, behavioral organization, mouth posture, oral reflexes, and non-nutritive sucking. The scale is scored on a scale from 0 to 2, with a maximum score of 36. The transition cut-off point for preterm infants to oral feeding has been established as 30 (16). The Turkish version of this scale, which was validated by Çamur and Çetinkaya, was found to have a cut-off point of 29 (17).

#### Sample size calculation

The sample size was calculated using PASS 2005 software (NCSS, Kaysville, UT, USA), found that 17 subjects were required for one group to achieve 90% power with a 5% type 1 error. To account for a potential 20% dropout rate, we recruited 20 subjects for each group, aiming to maintain 90% power in the study.

#### Statistical analysis

The test results were presented as mean ± standard deviation, median, and minimum-maximum values. To decide on the appropriate statistical methods for comparing the study groups, the homogeneity (Levene's Test) and normality (Shapiro-Wilk) tests were used. If the groups were normally distributed and exhibited homogeneous variances, comparisons between two groups were conducted using Student's *t*-test, while comparisons within dependent groups were done using the Paired *t*-test. However, some variables did not meet the parametric test assumptions, so comparisons between two independent groups were performed using the Mann-Whitney *U* test, and comparisons within dependent groups were conducted using the Wilcoxon test. For categorical data analysis, Fischer's Exact Test and Chi-square test were employed. When the number of cases was expected to be less than 20% of cells for inclusion in the analysis, the “Monte Carlo Simulation Method” was used to determine the values. All statistical analyses were carried out using SPSS software (IBM Corp. Released 2017. IBM SPSS Statistics for Windows, Version 25.0 Armonk, NY: IBM Corp.). A *p*-value of < 0.05 was considered statistically significant.

## Results

Forty infants (20 infants in oral-motor therapy group) were enrolled in the study. Mean gestational age and birth weights were 28.9 ± 1.3, 1302.7 ± 310.9 respectively. There were no statistically difference between oromotor intervention group and control group in terms of gestational age, birth weight, gender, 1st and 5th minute Apgar scores, maternal age, gravida, multiparity, small for gestational age, respiratory distress syndrome, intraventricular hemorrhage, and CRIB-2 score (*p* > 0.05) (Table 1).

**Table 1:** Clinical characteristics of the infants.

	Oral-motor therapy group *n* = 20	Control group *n* = 20	*P* value
Gestational age (weeks)	28.9 ± 1.3 (26.5–30.4)	28.9 ± 1.3 (26.2–30.6)	0.904
Birth weight (grams)	1302.7 ± 310.9 (680–2000)	1226.0 ± 308.5 (700–2055)	0.327
Girls *n* (%)	8 (40)	10 (50)	0.525
APGAR score			
1st min	5.0 ± 1.5 (2–7)	5.3 ± 1.2 (3–7)	0.659
5th min	3.7 ± 3.3 (1–8)	4.7 ± 3.2 (1–8)	0.495
Maternal Age (years)	31.3 ± 6.1 (24–42)	30.8 ± 6.8 (18–41)	0.968
Gravida (median)	3.0	3.0	0.883
CRIB-2 score	5.0 ± 3.9 (0–11)	7.3 ± 1.9 (3–12)	0.121
Twin Pregnancy *n* (%)	4 (20)	8 (40)	0.168
RDS *n* (%)	8 (40)	10 (50)	0.227
AGA *n* (%)	20 (100)	19 (95)	0.663
SGA *n* (%)		1 (5)	
IVH *n* (%)			
Grade 1	9 (45)	7 (35)	0.603
Grade 2	2 (10)	1 (5)	

AGA, appropriate for gestational age; CRIB, critical risk index for babies; IVH, intraventricular hemorhage; RDS, respiratory distress syndrome; SGA, small for gestational age.

Values are given average ± SD (min-max).

Daily weight gain, day at the NICU and type of diet at NICU stay were similar between the oral motor therapy and control group. Bottle feeding at discharge was higher, and OG tube feeding was lower in infants who received oral-motor therapy. Also, more infants breastfed after discharge in the intervention group (*p* < 0.05). EFS and POFRAS scores were statistically significant in the oral motor therapy group rather than the control group (*p* < 0.05) (Table 2).

**Table 2:** Clinical and feeding skill results.

	Oral-motor therapy group *n* = 20		Control group *n* = 20		*P* value
Start age of the infants for the groups (weeks)	31.5 ± 0.92 (29.4–33.4)		31.5 ± 0.63 (31.0–33.3)		0.341
Evaluation age of the infants after intervention (weeks)	35.57 ± 0.92 (33.4–37.4)		35.45 ± 0.70 (34.2–37.3)		0.297
EFS score	49.5 ± 7.2 (35–57)		36.3 ± 8.7 (25–53)		0.000*
POFRAS score	32.6 ± 3.0 (27–36)		21.6 ± 6.3 (11–32)		0.000*
Time to full oral feeding (corrected age- weeks)	37.4 ± 4.3 (30.7–44.7)		38.2 ± 4.0 (33.0–49.2)		0.58
Daily weight gain (grams)	19.0 ± 4.0 (12.1–27.9)		19.6 ± 4.9 (7.9–26.9)		0.71
Length of stay in NICU (days)	53.8 ± 24.5 (19–104)		56.7 ± 5.2 (29–97)		0.862
	*n*	%	*n*	%	
Type of diet at NICU					0.647
Breastmilk	15	75	13	65	
Formula	2	10	4	20	
Mixed	3	15	3	15	
Feeding type at discharge					
OG tube	8	40	16	80	0.024*
Bottle feeding	11	55	4	20	
Breast feeding	1	5	0	0	
Transition to breast feeding after discharge					
Yes	12	60	4	20	0.010*
No	8	40	16	80	
Signs of feeding intolerance					
Yes	6	30	10	50	0.197
No	14	70	10	50	

* *p*<0.05.

EFS, early feeding skills assessment tool; NICU, neonatal intensive care unit; OG, orogastric tube; POFRAS, preterm oral feeding readiness scale.

Values are given average ± SD (min-max).

## Discussion

In our study, we examined the effect of the oromotor therapy program in the NICU on the performance of transition to BF and feeding skills in preterm infants with feeding intolerance. The oromotor therapy group showed better oral-motor skills, transition to bottle feeding at discharge, and a better transition to BF after discharge.

Based on a systematic review of relevant studies (12), it was found that the implementation of oral-motor therapy for preterm infants in the NICU yields favorable outcomes.

Ostadi et al. (18) recruited 45 infants (average gestational age 28.5 weeks, birth weight 1,193 g) in NICU into three groups: Infants receiving non-nutritive sucking (NNS) exercise, infants receiving NNS and swallowing exercise (SE) and control group. They have shown that babies receiving either NNS or NNS + SE had less tube feeding on discharge and their POFRAS scores were better when compared to non-intervention controls. Oral feeding needs the coordination of sucking-swallowing-breathing (19). The average time for a coordinated sucking is approximately 34 weeks for preterms. This time can be delayed in cases of extreme prematurity, neurological problems and in babies with BPD (19). The findings of a meta-analysis indicate that non-nutritive sucking (NNS) accelerates the time to achieve full oral feeding, while sensorimotor interventions show potential for enhancing the sucking process (20). As per the advice of the ESPGHAN Committee on Nutrition and Invited Experts, non-nutritive sucking (NNS) before commencing oral feeding has been associated with reduced time to achieve full oral feeding and shorter hospital stays (Level of evidence: 3) (19). Wen-Si Ni et al. (21) applied sensory stimulation around the mouth and pressure involving the whole body to premature babies born under 34 weeks and weighing between 1,000–2,000 g, 24 h after birth. Consequently, the therapy group exhibited earlier transitions to oral feeding, shorter hospital stays, and a lower incidence of extrauterine developmental delay compared to the control group. Hwang et al. (22) and Arora et al. (23) used the PIOMI (Premature Infant Oral Motor Intervention), developed by Beckman, aims to activate muscle contraction and enhance strength by improving the functional response to pressure, movement, and control of lip, cheek, jaw, and tongue movements. They found improvements in feeding performance of infants better than control group. Hwang et al. (22), Bala et al. (24), Fucile et al. (25), Aguilar-Rodríguez et al. (26) evaluated oral feeding skills, and Arora et al. (23), Fucile et al. (27) evaluated motor function of infants in their studies. These studies also reported positive effects of oral-motor therapy in preterm infants in the NICU. Despite some studies not providing specific details about the stimulation program, oromotor therapy demonstrated beneficial effects on feeding skills in infants. Our findings align with the results of previous studies. Our EFS and POFRAS scores showed significant results in intervention group.

Hwang et al. (22), Arora et al. (23), and Fucile et al. (27) found different results in growth parameters of the infants. In our study, although, oromotor therapy enhanced feeding skills no significant difference in daily weight gain was observed between two groups. This may be because the intervention didn't change the daily milk intake of the infants and they got their daily intake via OG tube or bottle feeding.

While several studies have reported improved transition times from tube feeding to oral feeding (23, 26–29), our study did not find any significant difference between the groups in terms of the transition time to full enteral feeding.The comparable duration of transition observed between the two groups could potentially be attributed to the maturation of sucking developmental stages, which plays a crucial role in the enhancement of infantile sucking abilities. Another plausible factor could be the implementation of our NICU protocol, which emphasizes an optimized feeding procedure and early initiation of Kangaroo care practices. In a study conducted by Kim et al. (30), enteral nutrition development massage was administered to infants born before 34 weeks of gestational age for two weeks. The findings indicated that infants in the massage group exhibited an earlier transition to full enteral feeding, a notable increase in superior mesenteric artery blood flow, and accelerated growth compared to the control group. Tekgündüz et al. (31) performed abdominal massage to prevent feeding intolerance in premature infants born between 28 and 32 weeks. Consequently, the application of abdominal massage in enterally-fed infants yielded positive outcomes, as reported in terms of daily weight gain, vomiting frequency, abdominal circumference measurement, and gastric residual volume. Furthermore, it is worth considering that the combination of oromotor therapy with other types of massage may have synergistic effects on gastric motility and growth parameters, providing additional benefits to the infants.

Among the studies that assessed hospital stays, three (22, 23, 26) reported a significant decrease, while the remaining studies (27, 29) did not show relevant differences in this regard. In our study, we did not observe any significant difference in hospital stay between the two groups. Similar daily weight gains, and standard NICU protocol for discharge regardless of feeding type may be the explanation of this result in our study.

In the systemic review of Rodriguez Gonzalez P (12), oral sensorimotor stimulation did not reveal superior results on the infants' physiological data and breastfeeding skills. However, in our study, infants in the study group significantly differed in transferring to the mother's breast after discharge, even if they were not discharged. The assessment of the long-term effects of the intervention plan was only conducted in the study of Fucile et al. (27), which revealed that the subjects continued breastfeeding. Similar to Fucile's study, we conducted a follow-up assessment on infants to determine the duration required for transitioning to full enteral feeding via an OG-tube, as well as their subsequent transition to breastfeeding following discharge. Although most of the infants in the intervention group were discharged with bottle feeding, and those in the control group with OG-tube feeding, we maintained regular contact with families on a weekly basis to monitor the feeding skills of the infants. It is suggested that future studies incorporate an assessment of this aspect. This observation supports the notion that the benefits of therapy are not limited to the short term but continue to be sustained in the long term.

There were limitations on a study basis. In our study, there were test batteries evaluating feeding skills like POFRAS and EFS, but there were no evaluations involving nutritional performance. Further studies can be performed in nutritional performance of the infants including the amount of nutrition-related performance parameters such as suck-swallow-respiratory coordination, milk intake rate and sucking freaquency in a minute. Furthermore, the scope of this research is restricted by its inclusion of medically vulnerable preterm infants, among whom potential confounding medical complexities linked to neurobehavioral status may arise. While medical factors did not exhibit variances among the groups in our study, forthcoming studies could enhance the isolation of intervention effects by focusing on low-risk preterm infants.

In conclusion, oral-motor therapy used in the NICU has beneficial effects on feeding skills and transition to breastfeeding performance of early preterm infants. Notably, the intervention program has been observed to lack contraindications or adverse side effects, both during the course of the program and upon its completion. Moreover, it is noteworthy to emphasize the cost-effectiveness of this intervention program, as it does not require the utilization of specialized devices but instead relies on the expertise of a physiotherapist. This would improve the quality of care for early preterm infants, without a high economic impact on the NICU. In this study, despite no significant difference in the transition time to full oral feeds between the groups, the study group exhibited noteworthy outcomes, including variations in feeding type at discharge, the type of diet at discharge, and the success of transitioning to breastfeeding. We can conclude that the oromotor therapy in NICU improves the quality of sucking and contributes to the development of better oral-motor skills.

## Data Availability

The raw data supporting the conclusions of this article will be made available by the authors, without undue reservation.
